# Recent sex work and associations with psychosocial outcomes among women living with HIV: findings from a longitudinal Canadian cohort study

**DOI:** 10.1002/jia2.25874

**Published:** 2022-03-22

**Authors:** Carmen H. Logie, Nina Sokolovic, Mina Kazemi, Stephanie Smith, Shaz Islam, Melanie Lee, Rebecca Gormley, Angela Kaida, Alexandra de Pokomandy, Mona Loutfy, the CHIWOS Research Team

**Affiliations:** ^1^ Factor‐Inwentash Faculty of Social Work University of Toronto Toronto Ontario Canada; ^2^ Women's College Research Institute Women's College Hospital, Toronto, Ontario, Canada and United Nations University Institute for Water, Environment and Health Hamilton Canada; ^3^ Ontario Institute for Studies in Education University of Toronto Toronto Ontario Canada; ^4^ Alliance for South Asian AIDS Prevention Toronto Ontario Canada; ^5^ Faculty of Health Sciences Simon Fraser University Burnaby British Columbia Canada; ^6^ BC Centre for Excellence in HIV/AIDS Vancouver British Columbia Canada; ^7^ Department of Family Medicine McGill University Montreal Quebec Canada; ^8^ Chronic Viral Illness Service McGill University Health Centre Montreal Quebec Canada; ^9^ Faculty of Medicine University of Toronto Toronto Ontario Canada

**Keywords:** women, sex work, depression, injection drug use, racism, alcohol use

## Abstract

**Introduction:**

Sex workers are disproportionately impacted by the HIV pandemic across global contexts, in part due to social and structural contexts of stigma and criminalization. Among women living with HIV, there is a dearth of longitudinal information regarding dynamics of sex work engagement and associated social and health outcomes. In order to better understand the social contexts and health needs of sex working women living with HIV, this study aimed to understand recent sex work prevalence and its longitudinal associations with stigma, psychosocial and clinical HIV outcomes among women living with HIV in Canada.

**Methods:**

We conducted a three‐wave prospective cohort survey at 18‐month intervals with women living with HIV aged 16 and older in three Canadian provinces between 2013 and 2018. We used generalized estimating equations to examine longitudinal associations between recent (past 6‐month) sex work with three types of outcomes: psychosocial (recent violence, recent injection drug use, hazardous alcohol use, clinical depression and post‐traumatic stress disorder), clinical HIV (CD4 count and viral load) and stigma (HIV‐related stigma, racial discrimination and gender discrimination). Equations were adjusted for socio‐demographic factors associated with sex work across all three waves: province, age, income, gender identity, sexual orientation, education level, ethnicity and housing security.

**Results and Discussion:**

Of 1422 participants, 129 (9.1%) reported recent sex work during at least one wave (82 at baseline, 73 at first follow‐up and 32 at second follow‐up). In adjusted analyses, recent sex work was associated with psychosocial outcomes, including: past 3‐month violence (adjusted odds ratio [AOR] = 2.47, 95% CI = 1.70, 3.60), past 6‐month injection drug use (AOR = 3.49, 95% CI = 2.21–5.52), hazardous alcohol use (AOR = 2.00, 95% CI = 1.04–3.89) and depression (AOR = 1.51, 95% CI = 1.06–2.15). In unadjusted analyses, sex work was also associated with clinical HIV outcomes and gender discrimination, but not racial discrimination/HIV‐related stigma.

**Conclusions:**

Among women living with HIV in Canada, sex work engagement is dynamic, and sex workers are more likely to report recent violence, recent injection drug use, problematic alcohol use and clinical depression. Violence prevention and support, harm reduction, mental health promotion and sex work‐affirming programs could be employed to optimize health and rights for sex working women living with HIV.

## 1 INTRODUCTION

Globally, cisgender women sex workers have a 13‐fold increased odds of HIV infection than non‐sex working counterparts [1] and an overall HIV prevalence of 10.4% (95% CI 9.5–11.5) [2]. Sex workers experience marginalization due to social and structural contexts of stigma and criminalization [3, 4, 5, 6] that elevate exposure to violence [7], arrests and incarceration [3, 8]. Stigma is intersectional [9], meaning that stigma towards sex workers can intersect with stigma towards other socially marginalized identities, including gender, race and HIV‐positive serostatus [10, 11].

Social and health inequities may be exacerbated for sex workers living with HIV. A global review of sex workers living with HIV reported varying prevalence of antiretroviral therapy retention and viral load suppression [2]. A meta‐analysis of 29 studies that categorized “hard to reach” persons living with HIV as including sex workers, homeless individuals and people who used drugs reported that this “hard to reach” status was associated with reduced odds of optimal (>95%) ART adherence [12]. In a systematic review of 10 studies with sex workers living with HIV in Sub‐Saharan Africa, factors such as stigma and substance use were associated with lower linkage to, and retention in, care and lower antiretroviral therapy initiation [13]. In high‐income contexts, sex workers may also experience social disparities; for instance, a longitudinal study in Vancouver, Canada found that sex workers living with HIV reported a higher prevalence of food insecurity than HIV‐uninfected sex workers [14].

Intersecting stigmas [9], including HIV‐related stigma, racial discrimination, and gender discrimination, are understudied among sex workers living with HIV, yet associated with poorer quality of life [15] among women living with HIV (WLWH) in cross‐sectional studies in Canada. To better understand the social contexts and health needs of sex workers living with HIV, we examined longitudinal associations between recent sex work and stigma, psychosocial and clinical HIV outcomes among a cohort of WLWH in Canada.

## 2 METHODS

### 2.1 Study setting and population

This analysis draws on longitudinal survey data collected at 18‐month intervals between 2013 and 2018 as part of the Canadian HIV Women's Sexual & Reproductive Health Cohort Study (CHIWOS) in Ontario, Quebec and British Columbia, Canada [16, 17]. Peer Research Associates (PRAs) recruited WLWH aged 16 years or older using purposive sampling methods (e.g. word‐of‐mouth) and venue‐based recruitment (e.g. HIV clinics) [18, 19].

### 2.2 Data collection

At each wave, PRAs administered surveys in‐person, or by Skype or phone for participants in rural locations, using a web‐based interface. Surveys lasted an average of 90–120 minutes. PRAs re‐contacted individuals for follow‐up study visits.

We collected data on recent (past 6‐month) sex work and psychosocial (violence, injection drug use, hazardous alcohol use, clinical depression and post‐traumatic stress disorder [PTSD]), HIV clinical (CD4 count and viral load) and stigma (HIV‐related stigma, gender discrimination and racial discrimination) outcomes. We assessed socio‐demographic variables, including age, education (high school diploma or higher vs. no high school diploma), sexual orientation (lesbian, gay, bisexual or queer [LGBQ] vs. heterosexual), ethnicity (Indigenous, Black, White and other), marital status (married, single, divorced/widowed/separated and other), housing insecurity (dichotomous [yes/no]: monthly ability to pay for rent/mortgage) and gross annual household income (<$20,000 CAD, $20,000–40,000 CAD and >$40,000 CAD).

*Recent sex work* was assessed with the question: “In the past 6‐months, have you been provided with any of the following in exchange for sex? (money, drugs, shelter, food, gifts, clothes, services).”

To assess psychosocial outcomes, we asked participants whether they had: experienced any form of physical, verbal, controlling or sexual *violence* (yes/no) in the past 3 months; and/or used *injection drugs* (yes/no) in the past 6 months. We used the 3‐item Audit‐C scale to assess *alcohol use*, with a cut‐off score of 8 or higher indicating hazardous alcohol use (current study Cronbach's alpha = 0.57) [20]; the low reliability may be due to the small number of items [21, 22]. We assessed *clinical depression* using the Center for Epidemiologic Studies Depression 10‐item Scale (CES‐D 10) dichotomized with a cut‐off score of 10 or higher indicating probable depression (current study Cronbach's alpha = 0.87) [23]. We assessed *PTSD* (Waves 1 and 3 only) with the 6‐item PTSD Checklist Civilian Form (PCL‐C) with a cut‐off of 14 or higher indicating PTSD (current study Cronbach's alpha = 0.91) [24].

We assessed HIV clinical outcomes by asking participants to report their latest *CD4 count* (categorized into <500 or >500 cells/mm^3^) and whether their most recent *viral load* was detectable (>50 copies/ml; dichotomous: yes/no). These responses were validated against a subsample of participants (*n* = 356) whose data were linked with laboratory data, where we found high positive prediction (93.7, 95% confidence interval [CI]: 90.2–96.2) and negative prediction (80.4, 95% CI: 66.9–90.2) values [25].

We used the 10‐item HIV Stigma Scale (HSS)‐Revised to measure *HIV‐related stigma*, including personalized stigma, disclosure concerns, negative self‐image and public attitudes (current study Cronbach's alpha = 0.85) [26]. To assess *racial discrimination* (current study Cronbach's alpha = 0.96) and *gender discriminatio*n (current study Cronbach's alpha = 0.94), we used the 8‐item version of the Everyday Discrimination Scale (EDD) [27]. Gender and racial discrimination were only assessed in Waves 1 and 3, not at Wave 2.

### 2.3 Statistical analyses

We first conducted descriptive statistics, independent sample *t*‐tests and chi‐square analyses to compare participant characteristics by recent sex work at each wave. Outcome variables significantly associated with sex work during at least one wave of data in bivariate analyses were considered for further analyses. We used generalized estimating equations (GEE) with an exchangeable correlation matrix (assuming equal correlations between observations within person) and robust standard errors (reduces risk of error due to mis‐specified matrix) to examine longitudinal associations between recent sex work (measured three times) with outcomes across the three survey waves [28, 29]. At each wave, equations were adjusted for socio‐demographic factors (province, age, education level, sexual orientation, gender identity, ethnicity, housing security and income) associated with recent sex work at baseline. Prefer not to answer/don't know responses were treated as missing. We used multiple imputation to impute missing covariates by wave to ensure the comparability of unadjusted and adjusted odds ratios [30]. We did not impute sex work or outcome variables; the GEE model maximizes the use of available information by including all participants that had non‐missing data for each variable for at least one timepoint.

We conducted a sensitivity check by re‐running analyses considering only participants who participated in all three waves of data collection. Analyses were conducted in Stata Version 15 (College Station, TX, USA) [31]. All *p* values are two‐sided and significant at the 0.05 level.

### 2.4 Ethical considerations

All participants provided informed consent before commencing the interview. Research ethics board (REB) approval was provided by Women's College Hospital, University of Toronto, Simon Fraser University and the University of British Columbia/Providence Health, and McGill University Health Centre. Study sites with independent REBs also obtained their own approval prior to commencing enrolment. Participants received $50 CAD for participation in each survey.

## 3 RESULTS AND DISCUSSION

In Wave 1 [W1], 1422 participants completed the survey; socio‐demographic characteristics of these participants are reported in Table 1. Of the total sample, 1244 participants were retained at Wave 2 [W2] and 938 at Wave 3 [W3]. A total of 129 (9.1%) individuals reported recent sex work during at least one of the three waves (W1: *n* = 82; W2: *n* = 73; and W3: *n* = 32). Among these, most indicated recent sex work during only one wave (W1 only, *n* = 42; W2 only, *n* = 30; and W3 only, *n* = 9), while relatively fewer reported sex work at two waves (W1 and W2, *n* = 25; W1 and W3, *n* = 5; W2 and W3, *n* = 8) or all three waves (*n* = 10). Women engaged in sex work at W1 were more likely to be lost to follow up (odds ratio [OR] = 2.08, *p* = 0.002). Notably, at W1, the survey logic only allowed recent sex work responses for those who reported consensual sex in the past 6 months (*n* = 664); those not reporting recent consensual sex were assumed not to have engaged in recent sex work.

**Table 1:** Socio‐demographic characteristics of women living with HIV participants who reported on sex work at baseline in the Canadian HIV Women's Sexual & Reproductive Health Cohort Study (CHIWOS)

	Sex work in the past 6 months (*N* = 82)	No sex work in the past 6 months (*N* = 1340)	Missing	Difference
Variables	Mean (SD) or *N* (%)	Mean (SD) or *N* (%)	(*N*)	*p*‐value
Province				<0.001
*British Columbia*	36 (44%)	320 (24%)		
*Ontario*	30 (37%)	687 (51%)		
*Quebec*	16 (19%)	333 (25%)		
Age	38.49 (8.82)	43.09 (10.67)		<0.001
Education – high school degree or higher	54 (68%)	1,134 (85%)	7	<0.001
Sexual orientation – LGBQ	26 (32%)	154 (12%)	5	<0.001
Gender identity – transgender woman	18 (22%)	36 (3%)	9	<0.001
Ethnicity				<0.001
*Indigenous*	32 (39%)	286 (21%)		
*Black, African and/or Caribbean*	4 (5%)	414 (31%)		
*White*	41 (50%)	543 (41%)		
*Other*	5 (6%)	97 (7%)		
Marital status			2	0.001
*Married*	16 (19.5%)	438 (33%)		
*Single*	57 (69.5%)	632 (47%)		
*Divorced/widowed/separated*	9 (11%)	262 (19.5%)		
*Other*	0 (0%)	6 (0.5%)		
Secure housing	58 (71%)	1,212 (91%)		<0.001
Household gross annual income (CAD)			23	0.009
*Less than $20,000/year*	63 (81%)	838 (64%)		
*$20,000–$40,000/year*	11 (14%)	273 (21%)		
*Greater than $40,000/year*	4 (5%)	190 (15%)		

Abbreviations: LGBQ, lesbian, gay, bisexual or queer; SD, standard deviation.

In bivariate analyses, sex work was not associated with HIV stigma at any wave, so this outcome was dropped from further analyses. Table 2 reports the prevalence of each outcome separated by recent sex work at that wave, for each of the three waves. Sex work, compared to no sex work, was linked consistently with increased recent violence (W1: 62% vs. 58%; W2: 64% vs. 22%; and W3: 60% vs. 26%), injection drug use (W1: 49% vs. 6%; W2: 41% vs. 7%; and W3: 59% vs. 8%), hazardous alcohol use (W1: 4% vs. 2%; W2: 30% vs. 4%; and W3: 15% vs. 4%) and depression (W1: 61% vs. 48%; W2: 79% vs. 55%; and W3: 71% vs. 45%). As also presented in Table 2, GEE model results revealed that on average across waves, in adjusted analyses, recent sex work was associated with increased odds of past 3‐month violence (adjusted odds ratio [AOR] = 2.47, 95% CI = 1.70, 3.60), past 6‐month injection drug use (AOR = 3.49, 95% CI = 2.21–5.52), hazardous alcohol use (AOR = 2.01, 95% CI = 1.04–3.89) and clinical depression (AOR = 1.51, 95% CI = 1.06–2.15).

**Table 2:** Generalized estimating equations: outcomes associated with recent sex involvement over time among women living with HIV in the Canadian CHIWOS study

		Frequency of outcome (*N*, %) for those engaged in recent sex work versus not				
	Total *N* used in analyses	Recent	Wave 2	Wave 3	Unadjusted odds ratio (OR) [95% CI]	Adjusted odds ratio (AOR) [95% CI]
Experience of violence (past 3 months)	1395	48 (62%) v. 723 (58%)	47 (64%) v. 236 (22%)	18 (60%) v. 197 (26%)	2.97* [2.11, 4.18]	2.47* [1.70, 3.60]
Injection drug use (past 6 months)	1416	39 (49%) v. 84 (6%)	29 (41%) v. 75 (7%)	19 (59%) v. 65 (8%)	3.29* [1.93, 5.62]	3.49* [2.21, 5.52]
Hazardous alcohol use	1400	3 (4%) v. 26 (2%)	18 (30%) v. 24 (4%)	3 (15%) v. 21 (4%)	2.68* [1.25, 5.75]	2.01* [1.04, 3.89]
Depression	1415	49 (61%) v. 615 (48%)	58 (79%) v. 605 (55%)	22 (71%) v. 353 (45%)	1.81* [1.33, 2.47]	1.51* [1.06, 2.15]
PTSD	1416	49 (60%) v. 619 (47%)	N/A	20 (62%) v. 363 (45%)	1.59* [1.08, 2.34]	1.37 [0.90, 2.09]
CD4 count <500 cells/mm^3^	1349	31 (55%) v. 432 (39%)	52 (75%) v. 674 (63%)	1 (6%) v. 116 (26%)	1.45* [1.02, 2.05]	1.18 [0.82, 1.71]
Detectable viral load (>50 copies/ml)	1383	21 (30%) v. 183 (15%)	5 (8%) v. 79 (8%)	4 (13%) v. 55 (7%)	1.66* [1.07, 2.58]	1.15 [0.74, 1.81]

	Total *N* used in analyses	Outcome (mean, SD) for those engaged in recent sex work versus not			Unadjusted Std. Beta (β) [95% CI]	Adjusted Std. Beta (β) [95% CI]
Racial discrimination	1417	21.9 (11.6) v. 18.9 (11.0)	N/A	18.4 (11.2) v. 16.9 (10.4)	0.18 [−0.01, 0.37]	0.11 [−0.05, 0.29]
Gender discrimination	1061	23.6 (11.0) v. 19.3 (10.0)	N/A	20.1 (10.8) v. 17.4 (9.2)	0.29* [0.07, 0.51]	0.09 [−0.12, 0.30]

Note: Adjusted odds ratio accounts for gender identity, sexual orientation, educational attainment, ethnicity and housing security.

Abbreviations: CI, confidence interval; PTSD, post‐traumatic stress disorder.

* *p* < 0.05.

Sensitivity analyses results revealed some differences when considering only participants who participated at all three waves of data collection (*N* = 887) relative to those who did not. Participants lost to follow‐up were more likely to identify as Indigenous (OR = 3.01, *p* < 0.001), transgender (OR = 1.84, *p* = 0.03), LGBQ (OR = 1.61, *p* = 0.003), report unstable housing (OR = 2.24, *p* < 0.001) and report an income <$20,000 CAD (OR = 1.50, *p* = 0.001); they were less likely to identify as Black (OR = 0.48, *p* = 0.03). Among consistent participants, results were replicated for recent violence (AOR = 1.84, 95% CI = 1.14, 2.98), injection drug use (AOR = 3.34, 95% CI = 1.84–6.04) and clinical depression (AOR = 1.72, 95% CI = 1.03–2.87), but the effect on hazardous alcohol use was marginal (AOR = 2.12, 95% CI = 0.98–4.63).

We found that recent sex work was associated with increased likelihood of violence, injection drug use, hazardous alcohol use and clinical depression in this longitudinal cohort of WLWH in Canada. Together, these findings signal the urgent need to improve the wellbeing of WLWH engaged in sex work, particularly regarding mental health, violence prevention and harm reduction needs [32]. Specifically, our findings can inform integrated health services with and for WLWH. Findings provide insight into co‐occurring social and health inequities (violence, substance use and depression) that signal the need for a syndemics lens [33, 34] with sex working WLWH. Syndemics framings offer possibilities for intervention design to address individual‐level (e.g. depression) and population‐level (e.g. gender‐based violence) phenomena [35].

Our finding that sex work was associated with injection drug use among WLWH in Canada aligns with prior research that documented elevated HIV exposure risks at the intersection of sex work and injection drug use due to potential transmission via condomless sex and contaminated syringes [36, 37, 38]. HIV vulnerabilities linked with sex work and injection drug use are exacerbated in wider risk environments that include exposure to violence [35, 36, 37]. Alcohol and other substance use can also be a coping strategy for stressful sex work environments [3, 10, 39, 40, 41].

Sex working WLWH are at the nexus of mental health disparities among sex workers [42, 43, 44] and WLWH [45, 46]. Our findings build on prior research in Vancouver that found sex workers report a disproportionate burden of mental health challenges; they also found that mental health challenges were higher among sex workers who used drugs [47]. Contexts of violence may contribute to the higher prevalence of depression among sex workers compared to non‐sex worker counterparts [42, 43, 44, 48]. Similarly, histories of violence contribute to mental health challenges among WLWH [43, 45].

Our study also had limitations. It was susceptible to recall bias (self‐report of CD4 and viral load) and selection bias (differential loss to follow‐up). Additionally, the study was limited by not asking about sex work stigma, place of sex work and not differentiating between self‐identified sex workers and persons engaged in transactional sex. Racial and gender discrimination and PTSD were only measured at two timepoints; thus, estimates may be less strong for these variables. Despite these limitations, our study addresses knowledge gaps regarding sex work among WLWH and psychosocial needs.

## 4 CONCLUSION

Sex work engagement among WLWH is dynamic and may fluctuate over time. Sex positive [49] and sex work affirming [50] approaches alongside community‐based support [51] may promote health and wellbeing among sex working WLWH. Findings point to the urgent need for sex work affirming [52], women‐centred HIV services [17, 50, 53] grounded in harm reduction principles to optimize health and rights for sex working WLWH.

## COMPETING INTERESTS

The authors declare no competing interests.

## AUTHOR CONTRIBUTIONS

CHL conceptualized the manuscript and led writing. NS conducted analyses and substantially contributed to writing. MK, SS, SI, MLee, RG, AK, AdP and ML contributed to data acquisition and edited the manuscript. AK, AdP and ML were principal investigators and acquired funding. All authors (CHL, NS, MK, SS, SI, MLee, RG, AK, AdP and ML) have read and approved the final manuscript.

## FUNDING

CHIWOS is funded by the Canadian Institutes of Health Research (CIHR), the CIHR Canadian HIV Trials Network (CTN 262), the Ontario HIV Treatment Network (OHTN) and the Academic Health Science Centres (AHSC) Alternative Funding Plans (AFP) Innovation Fund. AdP received support from Fonds de Recherche du Québec‐Santé (FRQS) (Chercheur‐boursier clinician‐Junior 1) and AK received salary support through a Tier 2 Canada Research Chair in Global HIV and Sexual and Reproductive Health. CHL's efforts were supported by the Canada Research Chairs Program, Canada Foundation for Innovation, and Ontario Ministry of Research and Innovation.

CHIWOS Research Team: Rahma Abdul‐Noor (Women's College Research Institute), Aranka Anema (Harvard Medical School), Jonathan Angel (Ottawa Hospital Research Institute), Dada Mamvula Bakombo (McGill University Health Centre), Fatimatou Barry (Women's College Research Institute), Greta Bauer (University of Western Ontario), Kerrigan Beaver (Women's College Research Institute), Marc Boucher (CHU Ste‐Justine), Isabelle Boucoiran (CHU Ste‐Justine), Jason Brophy (Children's Hospital of Eastern Ontario), Lori Brotto (University of British Columbia), Ann Burchell (St, Michael's Hospital), Claudette Cardinal (Simon Fraser University), Allison Carter (Kirby Institute), Lynne Cioppa (Women's College Research Institute), Tracey Conway (Women's College Research Institute), José Côté (Centre Hospitalier de l'Université de Montréal), Jasmine Cotnam (Canadian Aboriginal AIDS Network), Cori d'Ambrumenil (AIDS Vancouver Island), Janice Dayle, (McGill University Health Centre), Erin Ding (British Columbia Centre for Excellence in HIV/AIDS), Danièle Dubuc, (McGill University Health Centre), Janice Duddy (Pacific AIDS Network), Mylène Fernet (Université du Québec à Montréal), Annette Fraleigh (Women's College Research Institute), Peggy Frank (Simon Fraser University), Brenda Gagnier (Women's College Research Institute), Marilou Gagnon (University of Victoria), Jacqueline Gahagan (Dalhousie University), Claudine Gasingirwa (Women's College Research Institute), Nada Gataric (British Columbia Centre for Excellence in HIV/AIDS), Rebecca Gormley (British Columbia Centre for Excellence in HIV/AIDS), Saara Greene (McMaster University), Danielle Groleau (McGill University), Charlotte Guerlotté (COCQ‐SIDA), Trevor Hart (Ryerson University), Catherine Hankins (McGill University), Roula Hawa (Women's College Research Institute), Emily Heer (Alberta Health Services), Robert S. Hogg (Simon Fraser University), Terry Howard (Glasshouse Consultants), Shazia Islam (Women's College Research Institute), Joseph Jean‐Gilles (GAP‐VIES), Hermione Jefferis (AIDS Vancouver Island), Evin Jones (Pacific AIDS Network), Charu Kaushic (McMaster University), Mina Kazemi (Women's College Research Institute), Mary Kestler (Oak Tree Clinic BCWH), Maxime Kiboyogo (McGill University Health Centre), Marina Klein (McGill University Health Centre), Nadine Kronfli (McGill University Health Center), Gladys Kwaramba (Women's College Research Institute), Gary Lacasse (Canadian AIDS Society), Ashley Lacombe‐Duncan (University of Michigan), Melanie Lee (Simon Fraser University), Rebecca Lee (CIHR Canadian HIV Trials Network), Jenny Li (British Columbia Centre for Excellence in HIV/AIDS), Viviane Lima (British Columbia Centre for Excellence in HIV/AIDS), Elisa Lloyd‐Smith (Vancouver General Hospital), Carmen Logie (University of Toronto), Evelyn Maan (Oak Tree Clinic), Valérie Martel‐Lafrenière (Centre Hospitalier de l'Université de Montréal), Carrie Martin (Canadian Aboriginal AIDS Network), Renee Masching (Canadian Aboriginal AIDS Network), Lyne Massie (Université du Québec à Montréal), Melissa Medjuck (formerly of the Positive Women's Network), Brigitte Ménard (McGill University Health Centre), Cari L. Miller (formerly of Simon Fraser University), Judy Mitchell (Positive Living North), Gerardo Mondragon (British Columbia Centre for Excellence), Deborah Money (Faculty of Medicine at UBC), Ken Monteith (COCQ‐SIDA), Marvelous Muchenje (Women's Health in Women's Hands CHC), Florida Mukandamutsa (CASM), Mary Ndung'u (African Partnership Against AIDS), Valerie Nicholson (Simon Fraser University), Kelly O'Brien (University of Toronto), Nadia O'Brien (McGill University Health Centre and McGill University), Gina Ogilvie (University of British Columbia), Susanna Ogunnaike‐Cooke (Public Health Agency of Canada), Joanne Otis (Université du Québec à Montréal), Rebeccah Parry (Simon Fraser University), Sophie Patterson (Simon Fraser University), Angela Paul (Positive Living North), Doris Peltier (Canadian Aboriginal AIDS Network), Neora Pick (Oak Tree Clinic BCWH), Alie Pierre (McGill University Health Centre), Jeff Powis (Michael Garron Hospital), Karène Proulx‐Boucher (McGill University Health Centre), Corinna Quan (Windsor Regional Hospital), Jesleen Rana (Women's Health in Women's Hands CHC), Eric Roth (University of Victoria), Danielle Rouleau (Centre Hospitalier de l'Université de Montréal), Geneviève Rouleau (Centre Hospitalier de l'Université de Montréal), Sergio Rueda (Centre for Addiction and Metal Health), Kate Salters (British Columbia Centre for Excellence in HIV/AIDS), Margarite Sanchez (ViVA), Roger Sandre (Haven Clinic), Jacquie Sas (CIHR Canadian HIV Trials Network), Édénia Savoie (McGill University Health Centre), Paul Sereda (British Columbia Centre for Excellence in HIV/AIDS), Stephanie Smith (Women's College Research Institute), Marcie Summers (formerly of the Positive Women's Network), Wangari Tharao (Women's Health in Women's Hands CHC), Christina Tom (Simon Fraser University), Cécile Tremblay (Centre Hospitalier de l'Université de Montréal), Jason Trigg (British Columbia Centre for Excellence in HIV/AIDS), Sylvie Trottier (Centre Hospitalier Universitaire de Québec), Angela Underhill (Women's College Research Institute), Anne Wagner (Ryerson University), Sharon Walmsley (University Health Network), Clara Wang (British Columbia Centre for Excellence in HIV/AIDS), Kath Webster (Simon Fraser University), Wendy Wobeser (Queen's University), Denise Wozniak (Positive Living Society of British Columbia), Mark Yudin (St. Michael's Hospital), Wendy Zhang (British Columbia Centre for Excellence in HIV/AIDS) and Julia Zhu (British Columbia Centre for Excellence in HIV/AIDS). All other CHIWOS Research Team Members who wish to remain anonymous.

## Data Availability

Data are available from the Women's College Research Institute Women and HIV Research Program Data Access Coordinator for researchers and students who meet the criteria for access to confidential data. The current Data Access Coordinator is Angela Underhill and she can be reached at angela.underhill@wchospital.ca. The criteria for access to the confidential data includes 1) being added as a CHIWOS researcher or student to the research ethics board (REB) application and 2) signing a CHIWOS Data Sharing and Collaboration Agreement. The de‐identified data set cannot be publicly shared at this point as we do not have community or REB approval to do so. Co‐authorship is a requirement for data access as per the CHIWOS authorship policy (www.chiwos.ca) which includes the requirement that the ICMJE authorship criteria be met by all authors.
